# Deep learning segmentation of fibrous cap in intravascular optical coherence tomography images

**DOI:** 10.1038/s41598-024-55120-7

**Published:** 2024-02-22

**Authors:** Juhwan Lee, Justin N. Kim, Luis A. P. Dallan, Vladislav N. Zimin, Ammar Hoori, Neda S. Hassani, Mohamed H. E. Makhlouf, Giulio Guagliumi, Hiram G. Bezerra, David L. Wilson

**Affiliations:** 1 Department of Biomedical Engineering, Case Western Reserve University, Cleveland, OH 44106 USA; 2 Harrington Heart and Vascular Institute, University Hospitals Cleveland Medical Center, Cleveland, OH 44106 USA; 3 Brookdale University Hospital Medical Center, 1 Brookdale Plaza, Brooklyn, NY 11212 USA; 4 Cardiovascular Department, Innovation District, Galeazzi San’Ambrogio Hospital, Milan, Italy; 5 Interventional Cardiology Center, Heart and Vascular Institute, University of South Florida, Tampa, FL 33606 USA; 6 Department of Radiology, Case Western Reserve University, Cleveland, OH 44106 USA

**Keywords:** Intravascular optical coherence tomography, Fibrous cap, Thin-cap fibroatheroma, Deep learning, Segmentation, Fibrous cap thickness, Biomedical engineering, Cardiology, Interventional cardiology

## Abstract

Thin-cap fibroatheroma (TCFA) is a prominent risk factor for plaque rupture. Intravascular optical coherence tomography (IVOCT) enables identification of fibrous cap (FC), measurement of FC thicknesses, and assessment of plaque vulnerability. We developed a fully-automated deep learning method for FC segmentation. This study included 32,531 images across 227 pullbacks from two registries (TRANSFORM-OCT and UHCMC). Images were semi-automatically labeled using our OCTOPUS with expert editing using established guidelines. We employed preprocessing including guidewire shadow detection, lumen segmentation, pixel-shifting, and Gaussian filtering on raw IVOCT (*r,θ*) images. Data were augmented in a natural way by changing *θ* in spiral acquisitions and by changing intensity and noise values. We used a modified SegResNet and comparison networks to segment FCs. We employed transfer learning from our existing much larger, fully-labeled calcification IVOCT dataset to reduce deep-learning training. Postprocessing with a morphological operation enhanced segmentation performance. Overall, our method consistently delivered better FC segmentation results (Dice: 0.837 ± 0.012) than other deep-learning methods. Transfer learning reduced training time by 84% and reduced the need for more training samples. Our method showed a high level of generalizability, evidenced by highly-consistent segmentations across five-fold cross-validation (sensitivity: 85.0 ± 0.3%, Dice: 0.846 ± 0.011) and the held-out test (sensitivity: 84.9%, Dice: 0.816) sets. In addition, we found excellent agreement of FC thickness with ground truth (2.95 ± 20.73 µm), giving clinically insignificant bias. There was excellent reproducibility in pre- and post-stenting pullbacks (average FC angle: 200.9 ± 128.0°/202.0 ± 121.1°). Our fully automated, deep-learning FC segmentation method demonstrated excellent performance, generalizability, and reproducibility on multi-center datasets. It will be useful for multiple research purposes and potentially for planning stent deployments that avoid placing a stent edge over an FC.

## Introduction

Thin-cap fibroatheroma (TCFA) is widely recognized as a prominent risk factor for plaque rupture, a major contributor to acute coronary syndromes (ACS)^1,2^. TCFA is typically characterized by the presence of a large lipid pool covered by a thin fibrous cap (FC) (< 65 µm) and increased macrophage activity^1,3^. However, Kume et al. reported thicker cap measurements using intravascular optical coherence tomography (IVOCT), possibly due to tissue shrinkage during histology^4^. The consensus standard also recommends adjusting this threshold when applied to IVOCT images to account for potential tissue shrinkage (10–20%) during histopathologic processing^5^. Intravascular imaging techniques such as intravascular ultrasound (IVUS) and IVOCT enables the assessment of FC thickness. Nevertheless, caution should be exercised when interpreting IVUS findings due to insufficient resolution (150–200 µm) for reliable thickness measurement. In contrast, IVOCT offers superior axial resolution (12–18 µm)^6^, facilitating precise measurement of FC thickness, identification of TCFA, and determination of plaque vulnerability^5^.

Despite its advantages in assessing thin FC tissues, IVOCT presents significant limitations for real-time treatment planning and research in large data sets. First, an IVOCT pullback typically consists of more than 300 image frames, resulting in a data overload. The comprehensive manual analysis of coronary plaques necessitates meticulous consideration of image characteristics, leading to a time-consuming and labor-intensive process. Second, manual analysis of IVOCT images can be prone to high levels of inter- and intra-observer variability^5^. For instance, our research group reported intra- and inter-observer variabilities of ≤ 5% and 6%, respectively, among experienced cardiologists in detecting stent struts in IVOCT images^7^. Since coronary plaques exhibit less distinct features compared to stent struts, the variability in plaque analysis among clinicians is expected to be even higher. An automated method will be more reproducible which will be especially important if one is looking for changes between cohorts or within a cohort as with a drug trial. Third, manual point measurements of fibrous cap thickness do not fully capture tendency to rupture. Automated assessment of the surface area of a lesion is surely needed to assess vulnerability. These observations underscore the imperative for an accurate and fully-automated method for fibrous cap analysis.

There have been only a few studies addressing these limitations. Our group initially developed a semi-automated method for volumetric quantification of FC^8^. Briefly, we segmented the luminal and abluminal boundaries in the polar coordinates of IVOCT images using dynamic programming. Then, we quantified the thickness at each point of the FC luminal boundary. Although the method was validated in various ways, the manual identification of the circumferential distribution of the lipid was required. Zahnd et al.^9^ proposed a semi-automatic segmentation method utilizing dynamic programming to quantify coronary FC thickness in IVOCT images. The method was evaluated through multiple approaches, and the results were promising. However, its major limitation was the requirement of manual initialization of lipidic plaque for fibrous cap segmentation. Additionally, the method was validated on a small subset of pullbacks (179 images from 21 patients), which reduces its reproducibility. Min et al.^10^ developed a deep learning model using a DenseNet architecture for classifying IVOCT frames as either TCFA or non-TCFA. They reported a promising classification performance with an overall accuracy of 91.6 ± 1.7%, sensitivity of 88.7 ± 3.4%, and specificity of 91.8 ± 2.0%. However, this method did not offer quantitative measurements of FC. In our previous study^11^, we developed an automated method capable of detecting lipidic plaque and segmenting the FC in IVOCT images. The method consisted of two phases: lipidic plaque detection using deep learning and FC segmentation using dynamic programming. Evaluation was performed on over 4,000 image frames from 41 patients, demonstrating excellent discriminability of lipidic plaque and good reproducibility in FC thickness measurement. However, our previous method occasionally exhibited inaccurate lipid arc detections, affecting the accuracy of FC thickness measurement. An accurate and fully automated end-to-end training method could offer faster and improved assessment of FC.

In this report, we expand upon our previous study^11^ and develop a fully-automated deep learning method for FC segmentation in IVOCT images. To achieve this, we employ specialized image preprocessing, transfer learning, a large carefully labeled dataset, physics- and system-plausible augmentation, and advanced deep learning networks. Robustness, accuracy, and reproducibility of results are carefully evaluated. Because we are automatically analyzing volumes of data, we create visual heatmaps of fibrous cap thickness, giving a compelling visualization of vulnerability.

## Image analysis methods

### Preprocessing

**Figure 1:**
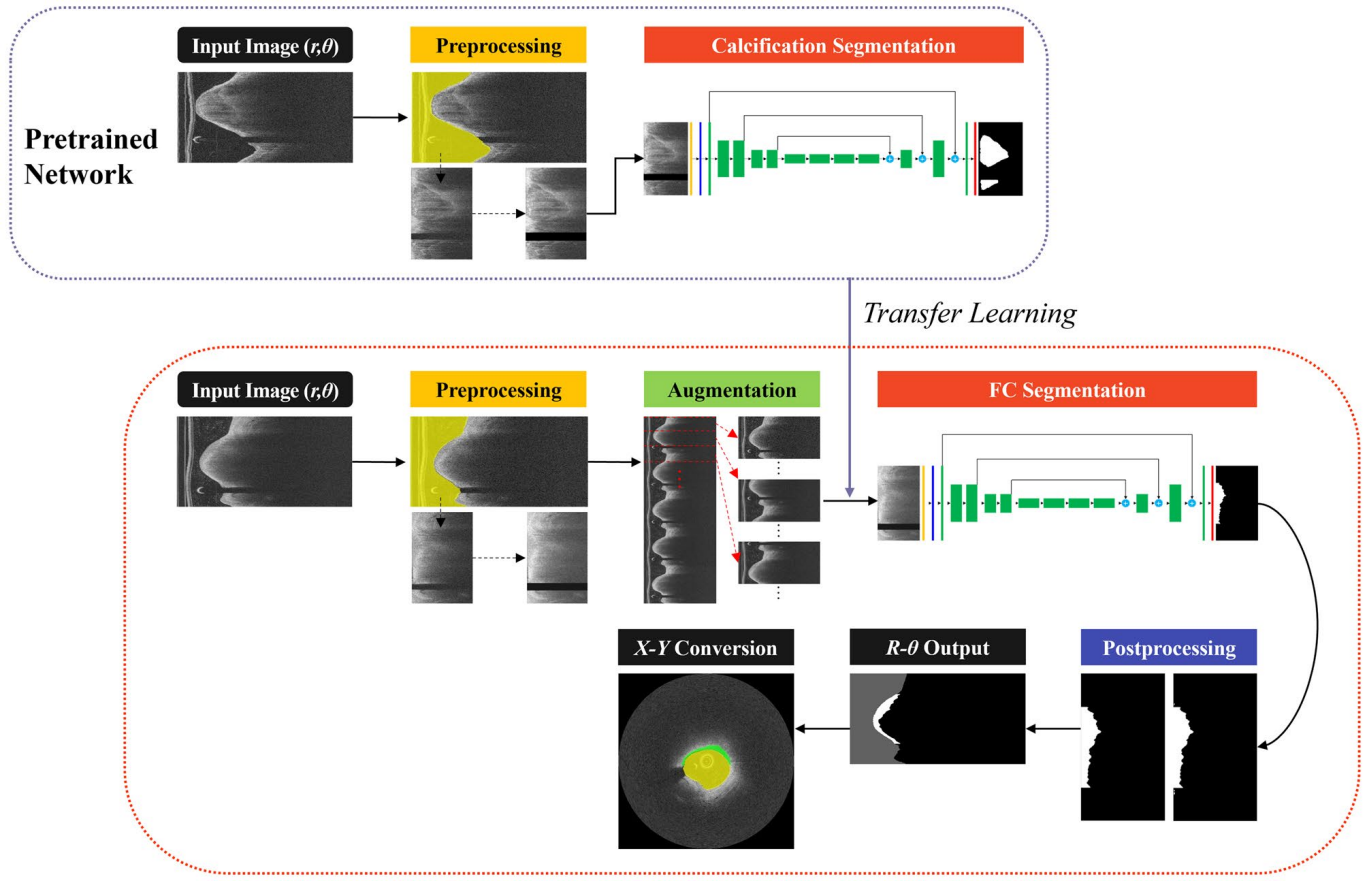
Workflow of FC segmentation in IVOCT images. The key steps include preprocessing, data augmentation, FC segmentation, transfer learning, and postprocessing. Preprocessing involves guidewire shadow detection, lumen segmentation, pixel-shifting, and noise filtering on raw IVOCT data (*r,θ*), followed by data augmentation on the preprocessed images. The output serves as input to the FC segmentation network. Postprocessing techniques, such as filling and morphological operations, are utilized to reduce small false positive errors. For transfer learning (top, blue dotted box), the network is trained using IVOCT calcification images with the same preprocessing and network architecture.

We employed a preprocessing method previously proposed by our group^12,13^ to identify the appropriate tissue regions of interest for FC segmentation. Preprocessing of raw IVOCT (*r,θ*) image data involved several steps: (1) Detection and removal of the guidewire and corresponding shadow regions using dynamic programming^14^, as they do not contain tissue information. (2) Segmentation of the lumen boundary using a deep learning-based semantic segmentation method developed by our group^15^. (3) Pixel-shifting each A-line to the left, ensuring that all A-lines have the same starting point along the radial direction. This step was crucial as it not only created a smaller region of interest for deep learning, simplifying the processing, but also aligned the tissues, making different lesions appear more similar to the network^11^. (4) Limiting the *r* direction to the first 200 pixels (~ 1 mm) due to the limited penetration depth of the IVOCT signal. (5) Applying a Gaussian filter with a kernel size of (7,7) and a standard deviation of 1-pixel to reduce noise. After preprocessing, the size of the IVOCT data was reduced from (968 × 448 pixels) to (200 × 448) without any loss of meaningful data. The preprocessed images were then used for further processing. The overall workflow of the proposed method is illustrated in Fig. 1. Please note that all images in the manuscript are presented following a log transformation for improved visualization.

### Data augmentation for deep learning

Our previous study found that data augmentation significantly improved deep learning segmentation performance in IVOCT images^15,16^. The IVOCT data were augmented for deep learning training to increase the number of examples with varying FC locations and intensities, thereby enhancing the spatial invariance of the methods. We applied two data augmentation approaches, one for the raw polar IVOCT before preprocessing and another for the preprocessed IVOCT images. First, for the raw polar IVOCT pullback, we concatenated all of the raw polar (*r*,*θ*) images to form one large 2D array, where *r* represents tissue depth and the *θ* is catheter rotation, which rotates from 0 to *N* × 360°, where *N* is the number of unput images. Then, we changed the offset angle to extract new polar image frames with no data loss or distortion. In practice, we shifted the starting A-line six times by 80 A-line increments. Further details are provided elsewhere^15,16^. Second, the data augmentation for preprocessed IVOCT data involved several steps as follows: (1) We normalized the intensity of all input images to the range (0, 1). (2) We flipped input images along the vertical axis with a 10% probability. (3) We randomly scaled the pixel intensity of the input image by a factor of 0.1 with a 20% probability. (4) We randomly shifted the pixel intensity of the input image by a factor of 0.1 with a 20% probability.

### FC segmentation

**Figure 2:**
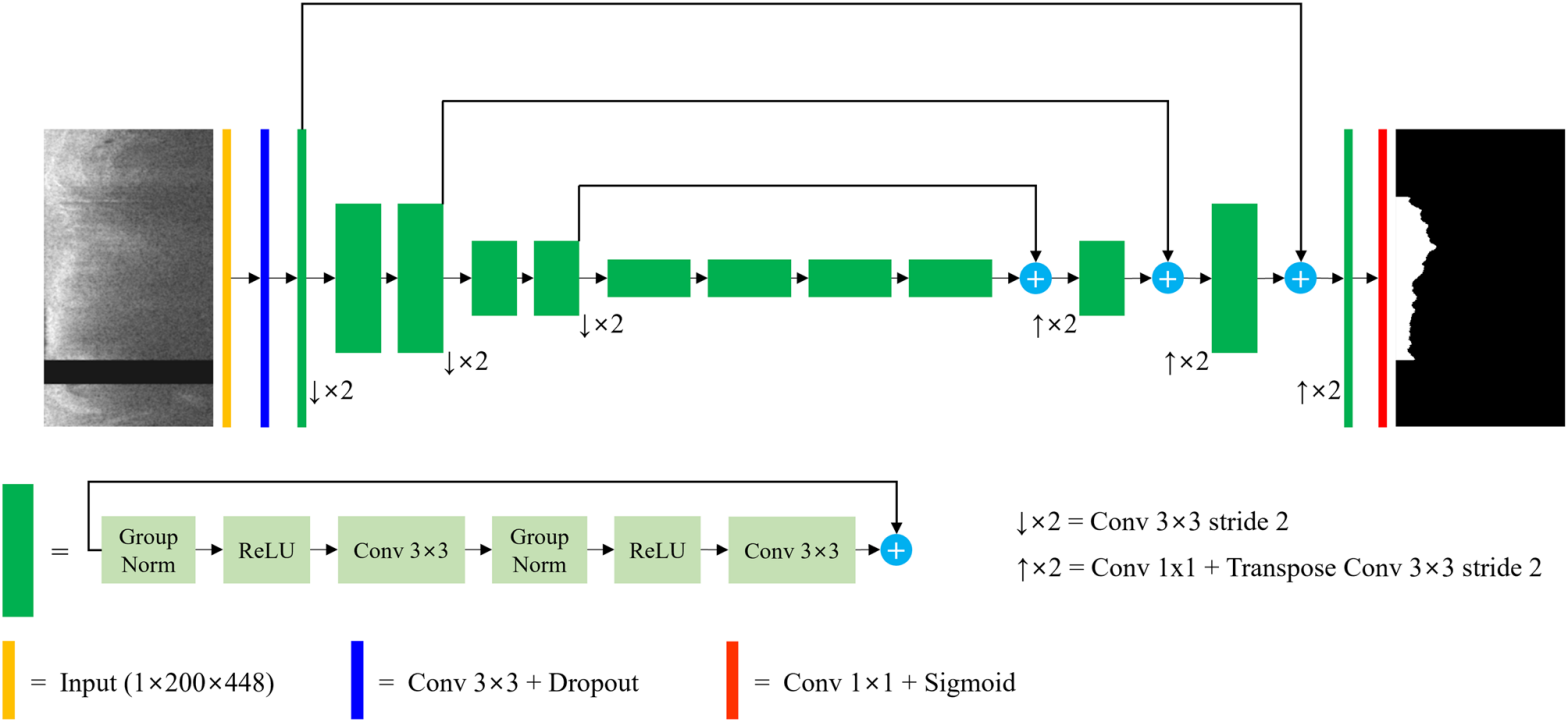
SegResNet Architecture for FC Segmentation. The preprocessed IVOCT image serves as the input, starting with an initial 3 × 3 convolution and dropout layers. Each green block represents a ResNet-like block with group normalization. The decoder outputs a predicted label, followed by a sigmoid activation function to generate a pixel-wise classification map. Both the input and output images have the same size (200 × 448 pixels in (*r,θ*)). In the input image, the black strip indicates the removed guidewire shadow.

To segment FC regions in IVOCT images, we utilized a modified version of SegResNet^17^, which follows an encoder-decoder-based convolutional neural network (CNN) architecture with an asymmetrically large encoder backbone and a smaller decoder (Fig. 2). In our study, the variational auto-encoder branch was excluded since we had a sufficient number of IVOCT image instances (> 32,000) for FC segmentation.

The encoder component primarily consisted of ResNet^18^ blocks, where each block comprised two convolutions with normalization and rectified linear unit activation, followed by an additive identity skip connection. For normalization, we employed Group Normalization^19^, which demonstrates improved performance compared to Batch Normalization when the batch size is small. We adopted a conventional CNN approach to progressively downsize the image dimensions by a factor of 2 while simultaneously increasing the feature size by 2. Strided convolutions were employed for downsizing, and all convolutions were 3 × 3 with an initial number of filters set to 16. Additionally, a dropout layer with a probability of 0.2 was incorporated into each block.

The decoder component resembled the encoder part but contained only a single block for each spatial level. Each decoder level commenced with upsizing, which reduced the number of features by a factor of 2 using 1 × 1 convolutions and doubled the spatial dimension via bilinear upsampling. Subsequently, the encoder output from the corresponding spatial level was added. The output of the decoder retained the same size as the original image, and the number of features matched the initial input feature size. Finally, a 1 × 1 convolution layer and a sigmoid function were applied.

### Transfer learning for deep learning

To optimize the deep learning training for FC segmentation, we implemented domain adaptation transfer learning using our existing calcification IVOCT dataset. The rationale behind employing transfer learning is to decrease training time and alleviate the requirement for a large number of training samples by leveraging a model previously trained on a different yet related task—specifically, calcification segmentation in IVOCT images. Domain adaptation serves to prevent or minimize negative migration during training by capitalizing on knowledge acquired from a source domain (calcification segmentation) to enhance performance on a target domain (FC segmentation). For instance, the lower-level features learned during the initial training (calcification) prove valuable for the target domain (FC). By reusing these features, the model can concentrate on learning task-specific details pertinent to FC segmentation without discarding valuable knowledge. Additionally, rather than training the entire model from scratch, which may lead to negative transfer, only specific layers or parameters of the pre-trained model are adjusted during adaptation to the target task. This approach enables the model to retain knowledge from the source domain while adapting to the specifics of the new task. For this study, we constructed a pretrained network with the same architecture as the FC segmentation model using the IVOCT data from our previous studies on calcification segmentation (comprising over 24,000 images)^13,15,20,21^. Rather than initializing the weights of the second network (FC segmentation) randomly, we utilized the pretrained model to initialize these weights. Through transfer learning, the model can rapidly reach the convergence point during training, potentially leading to enhanced performance.

### Postprocessing

To clean results and enhance segmentation performance, we implemented a morphological operation after the FC segmentation. Given the presence of inherent speckle noises in IVOCT images, the network occasionally exhibits spotty segmentation errors throughout the pullbacks. We employed an opening operation on the output labels with a disk-shaped structuring element with a radius of 3. Subsequently, we filled in the holes within the segmented labels. The pixel connectivity rule was set to 4.

## Experimental methods

### Data acquisition

The images utilized in this study were obtained from two sources: the TRiple Assessment of Neointima Stent FOrmation to Reabsorbable polyMer with Optical Coherence Tomography (TRANSFORM-OCT) trial^22^ and the University Hospitals Cleveland Medical Center (UHCMC) Registry. The TRANSFORM-OCT dataset comprised 24,209 images (15,239 calcification and 8,970 FC images) derived from 153 pullbacks involving 77 patients. On the other hand, the UHCMC dataset consisted of 8,322 images (6,960 calcification and 1,362 FC images) acquired from 74 pullbacks involving 74 patients. The raw IVOCT data size was 968 × 448 in the (*r,θ*) domain. Calcification images were employed to establish the pretrained network for transfer learning, whereas FC images were employed for training the network specifically for FC segmentation (Fig. 1). The IVOCT images were acquired using a frequency-domain ILUMIEN OCT system (Abbott Vascular, Santa Clara, CA, USA), which utilized a tunable light source sweeping from 1,250 to 1,360 nm. The imaging pullback was conducted at a frame rate of 180 fps, pullback speed of 36 mm/s, and an axial resolution of approximately 20 µm.

The inclusion criteria encompassed patients with stable angina and documented ischemia or acute coronary syndrome who had undergone IVOCT examination. Major exclusion criteria included the presence of unprotected left main disease, chronic total occlusion, baseline serum creatinine > 2.0 mg/dL, life expectancy < 18 months, and unsuitability for OCT imaging at the clinician's discretion. Additionally, IVOCT image frames with poor quality due to luminal blood, unclear lumen, artifact, and reverberation were excluded. IVOCT pullbacks with excessive side branches and previous stent implantation were also excluded. Some pullbacks had the presence of thrombus (40% for TRANSFORM and 24% for UHCMC), but the actual number of image frames was negligible, as only the frames with FC were selected. This study adhered to the principles outlined in the Declaration of Helsinki and received approval from the Institutional Review Board of University Hospitals Cleveland Medical Center, Cleveland, OH, USA. The requirement for individual informed consent was waived as all data were fully anonymized, with no identifiable personal health data.

### Ground truth labeling

The FC regions were segmented using Optical Coherence TOmography PlaqUe and Stent (OCTOPUS) software, previously developed by our group^23^, and manually edited by two experts (5 + years of experience) from the Cardiovascular Imaging Core Laboratory at University Hospitals Cleveland Medical Center, a leading IVOCT analysis laboratory with over 3,000 clinical trial cases analyzed. For manual editing of FC, we followed the “consensus document” for IVOCT image analysis^5^. Specifically, the FC was defined as a distinct tissue layer of connective tissue, which is often signal-rich, overlying a signal-poor region, and TCFA was defined as a necrotic core with an overlying fibrous cap where the minimum thickness of the fibrous cap was less than a predetermined threshold (< 65 µm). The labels provided by the more experienced expert were used as the ground truth. In case of disagreement between the two readers, they revaluated the frames and reached a consensus decision. Any region other than FC was given a label of “background”, which allowed us to set up a binary segmentation task.

### Network training

We utilized the AdamW optimizer, an adaptive moment estimation optimizer with decoupled weight decay^24^, for training both the transfer learning and FC segmentation networks. This optimizer employs stochastic gradient descent and adaptively estimates first-order and second-order moments while incorporating a weight decay method^24^. The AdamW optimizer is computationally efficient, robust to diagonal rescaling of gradients, and well-suited for handling large-scale data problems.

The initial learning rate, epsilon, and weight decay were empirically set to 1 × 10^–5^, 1 × 10^–9^, and 1 × 10^–6^, respectively. To train the networks, we employed a maximum of 600 epochs and a batch size of 64. L2 norm regularization with a weight of 1 × 10^–6^ was applied to the convolutional kernel parameters, and spatial dropout with a rate of 0.2 was implemented after the initial encoder convolution, following the original implementation^17^.

For the FC segmentation model, the learning parameters of each encoder layer were initialized by transferring weights from the transfer learning network, which was pretrained on IVOCT calcification data. The network weights were then fine-tuned in a layer-by-layer manner using backpropagation. The learning rates of subsequent layers were adjusted sequentially until the performance on the validation set ceased to improve.

The loss functions for both networks were computed using the Dice loss function over the softmax outputs. To prevent overfitting during training, we employed a stopping criterion that halted training when the validation loss failed to improve for 10 consecutive epochs or when the maximum number of epochs was reached. In practice, the former rule was executed. We used the following frameworks using Python (ver. 3.9.13, Python Software Foundation, USA): Pytorch (ver. 1.13.1) and Monai (ver. 1.1.0).

### Performance evaluation

For transfer learning training, we partitioned a total of 227 pullbacks into training and validation sets. Following a 7:3 split, the training set consisted of 15,239 calcification images from 153 pullbacks (TRANSFORM-OCT), while the validation set contained 6,960 calcification images from 74 pullbacks (UHCMC). There was no held-out test set for pretraining as the network was solely employed for transfer learning purposes.

Regarding FC segmentation, we performed a five-fold cross-validation on the TRANSFORM-OCT dataset, which encompassed 8,970 FC images from 153 pullbacks. In each fold, there were sub-groups for training (60%), validation (20%), and testing (20%). Folds and sub-groups were based on pullbacks rather than images. This approach ensured that each sub-group was assigned to the test set precisely once, thereby avoiding evaluation variance. Additionally, the UHCMC dataset, comprising 1,362 FC images from 74 pullbacks, served as the held-out test set for further evaluations.

The segmentation performance was quantitatively assessed using conventional metrics, including pixel-wise positive predictive value (PPV), negative predictive value (NPV), sensitivity, specificity, and Dice coefficient as below:

1 $${\text{PPV }} = {\text{ TP }}/ \, \left( {{\text{TP }} + {\text{ FP}}} \right)$$

2 $${\text{NPV }} = {\text{ TN }}/ \, \left( {{\text{TN }} + {\text{ FN}}} \right)$$

3 $${\text{Sensitivity }} = {\text{ TP }}/ \, \left( {{\text{TP }} + {\text{ FN}}} \right)$$

4 $${\text{Specificity }} = {\text{ TN }}/ \, \left( {{\text{TN }} + {\text{ FP}}} \right)$$

5 $${\text{Dice coefficient }} = {\text{ 2TP }}/ \, \left( {{\text{2TP }} + {\text{ FP }} + {\text{ FN}}} \right)$$

Here, TP represents the number of true positive pixels, TN denotes the number of true negatives, FP signifies the number of false positives, and FN represents the number of false negatives. We reported the mean and standard deviation of these metrics across the five folds. Furthermore, to investigate performance variance, we compared the segmentation results against three other networks (U-Net^25^, Attention U-Net^26^, and nnU-Net^27^). Training details for all deep learning networks are provided in Supplementary Table S1. We also examined the impact of transfer learning on the results by comparing performance with and without transfer learning. To conduct this analysis, we created pretrained networks for all four networks, utilizing the exact same calcification-analysis training and validation datasets. Additionally, we assessed the reproducibility of the proposed method by evaluating pre- and post-stenting IVOCT pullbacks acquired from the same lesion.

In addition to conventional metrics, we also assessed a clinically meaningful metric, specifically FC thickness, which has been utilized in several prior clinical research studies^28,29^. This metric was evaluated on the held-out test set. FC thickness is defined as the distance between the luminal and the abluminal boundaries. Measurements were taken along the abluminal boundary from the luminal boundary at each A-line in polar (*r,θ*) images. Subsequently, the mean FC thickness for each FC plaque region was determined by averaging these measurements.

## Results

**Figure 3:**
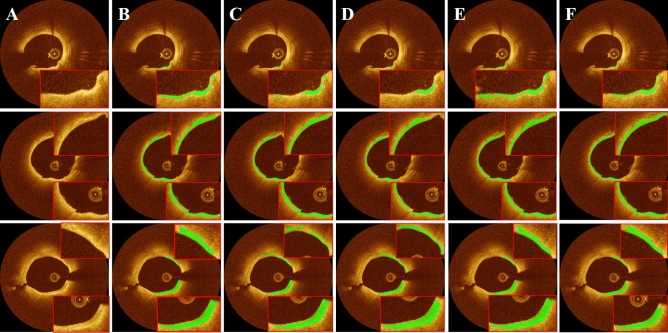
FC Segmentation results for different deep learning models with transfer learning. The panels include (**A**) IVOCT image in Cartesian coordinates, (**B**) ground truth, (**C**) U-Net, (**D**) Attention U-Net, (**E**) nnU-Net, and (**F**) SegResNet. Each row represents different instances of IVOCT images with FC present (shown in green). Among all the networks, SegResNet exhibited the highest segmentation performance in terms of Dice (0.837 ± 0.012) and PPV (82.5% ± 3.7%) across all five-folds of cross-validation. The green color indicates FC plaque regions, which are magnified within the red boxes.

For FC segmentation, SegResNet consistently delivered the most reliable segmentation results as compared to other deep learning networks (Fig. 3 and Table 1). SegResNet segmentations are nearly visually identical to manual labels (Fig. 3). Attention U-Net exhibited the lowest Dice coefficient (0.806 ± 0.022) and sensitivity (80.1% ± 4.9%) among all the networks (Table 1). The nnU-Net showed a slightly higher (or equivalent) sensitivity (91.4% ± 1.5%) and Dice coefficient (0.837 ± 0.008) than the SegResNet. However, its PPV was the lowest (77.2% ± 0.3%), indicating a higher rate of false positives. Overall, the SegResNet demonstrated the best segmentation performance out of all the networks employed. All networks underwent the same preprocessing, data augmentation, transfer learning, and postprocessing. As described previously, networks were pre-trained on the task of segmenting calcifications, using our large database of such images. For more information on pre-training, see Supplementary Fig. S1 and Table S2.

**Table 1:** Mean quantitative performance metrics of FC segmentation across five-folds for various deep learning networks, including U-Net, Attention U-Net, nnU-Net, and SegResNet. Among all the networks employed, SegResNet demonstrated the best segmentation results, achieving a Dice coefficient of 0.846 ± 0.011 and a PPV of 84.2% ± 1.8%.

	PPV (%)	NPV (%)	Sensitivity (%)	Specificity (%)	Accuracy (%)	Dice
U-Net	84.0 ± 1.8	98.9 ± 0.1	80.2 ± 0.1	99.1 ± 0.1	98.1 ± 0.0	0.820 ± 0.009
Attention U-Net	81.2 ± 0.6	98.9 ± 0.2	80.1 ± 4.9	98.9 ± 0.2	97.9 ± 0.1	0.806 ± 0.022
nnU-Net	77.2 ± 0.3	99.5 ± 0.1	91.4 ± 1.5	98.5 ± 0.1	98.1 ± 0.0	0.837 ± 0.008
SegResNet	84.2 ± 1.8	99.1 ± 0.0	85.0 ± 0.3	99.1 ± 0.1	98.3 ± 0.1	0.846 ± 0.011

**Figure 4:**
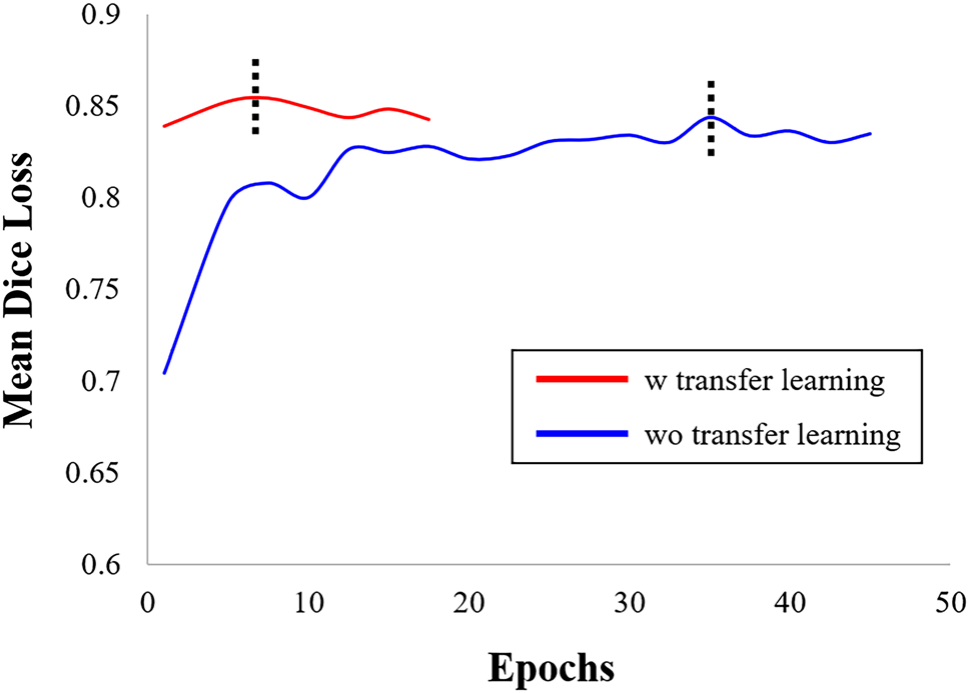
Mean Dice loss curve during validation as computed over a fold. Red and blue curves are results with and without transfer learning, as described in the text. With transfer learning, the curve reached an asymptotic result with many fewer epochs. In addition, there was an improved Dice value in this run with transfer learning. The black dotted lines indicate the points of highest Dice coefficients for each curve.

**Figure 5:**
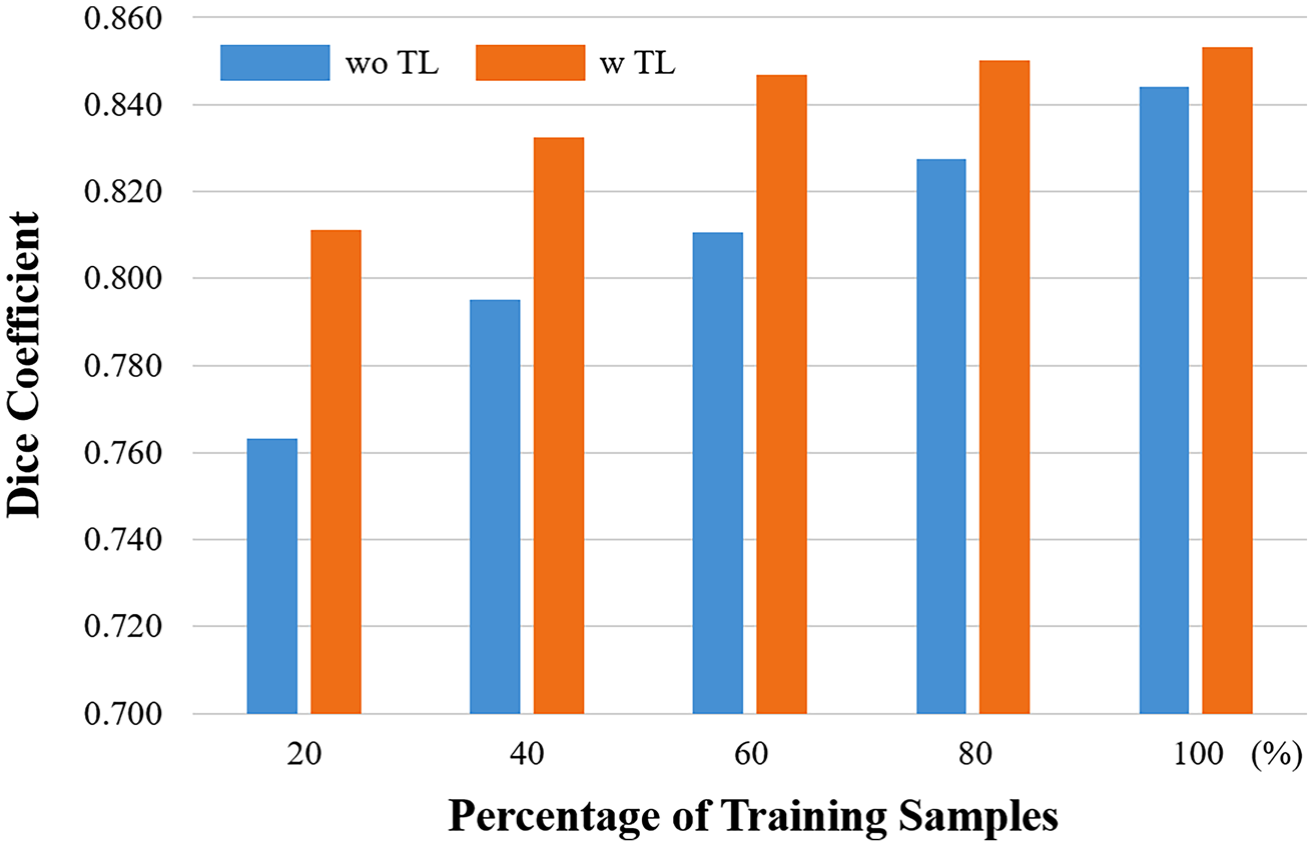
Effect of pretraining and transfer learning on the number of labeled samples required for training. With transfer learning (TL, brown bars), Dice values are always greater than the result without transfer learning. In addition, with transfer learning, there is convergence towards an asymptotic Dice value whereas without transfer learning, performance is continuing to improve much between 80 and 100% of labeled training samples. Note that with transfer learning, only 60% of samples gives a better result than 100% of the samples without transfer learning. The task for pretraining was segmentation of calcified plaques in IVOCT images.

Transfer learning from the calcification segmentation task reduced training time without yielding significant improvements in FC segmentation performance. Figure 4 shows mean validation Dice loss curves with and without transfer learning from SegResNet. Without transfer learning, convergence (as determined by the stopping rule) required approximately 22–42 epochs, whereas with transfer learning, convergence was achieved in only 3–7 epochs. Transfer learning also reduced the need for a large number of training samples (Fig. 5). With transfer learning, Dice values always exceed results without transfer learning. They appear to reach asymptotic convergence unlike without transfer learning. With transfer learning, only 60% of samples gives a better result than 100% of the samples without transfer learning. Using the full data set and long training times, transfer learning resulted in somewhat improved quantitative metrics across the folds for all networks, as shown in Supplementary Table S3. If we reduce the training samples by 40%, then there is a significant difference between with and without transfer learning (not shown).

**Figure 6:**
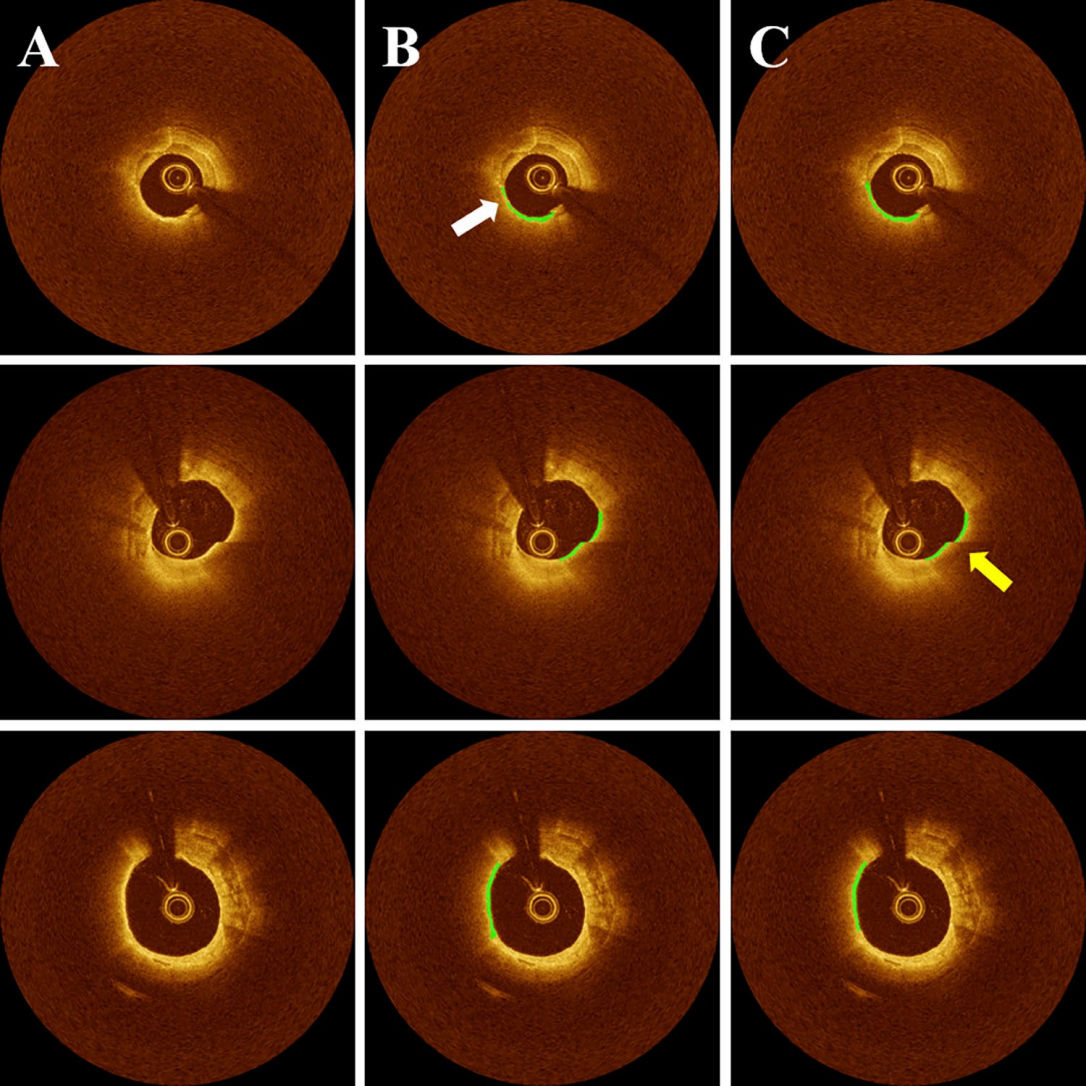
FC segmentation results on the held-out test set. The panels include (**A**) Cartesian IVOCT image, (**B**) ground truth, and (**C**) automated prediction. Each row represents different instances of IVOCT images. In panel (**B**) (top), the ground truth FC label appears disconnected at 8 o'clock (indicated by the white arrow); however, our proposed method accurately predicts FC regions, demonstrating its high generalizability. Moreover, our method delivered reliable results even in the presence of image reconstruction errors, as depicted in panel (**C**) (at 3 o'clock, highlighted by the yellow arrow). The green color indicates FC plaque regions.

Our method had highly consistent segmentation results between the five-fold cross-validation (TRANSFORM-OCT) and the held-out test (UHCMC) sets (Fig. 6 and Table 2). To achieve this, we applied each of the five trained models from the cross-validation on the held-out test set, which consisted of 1,362 FC images from 74 pullbacks at UHCMC. We combined the pixel-wise predictions by selecting the most common output through plurality voting. On the held-out set, the proposed method achieved a PPV of 78.5%, sensitivity of 84.9%, and Dice coefficient of 0.816. These metrics indicate a high level of generalizability for our method.

**Table 2:** Quantitative performance metrics of FC segmentation on the held-out test set, including PPV, NPV, sensitivity, specificity, accuracy, and Dice coefficient. Our method demonstrates highly consistent segmentation results between the cross-validation and held-out test sets, highlighting the remarkable generalizability of the proposed approach.

	PPV (%)	NPV (%)	Sensitivity (%)	Specificity (%)	Accuracy (%)	Dice
Our method	78.5	99.4	84.9	99.0	98.5	0.816

**Figure 7:**
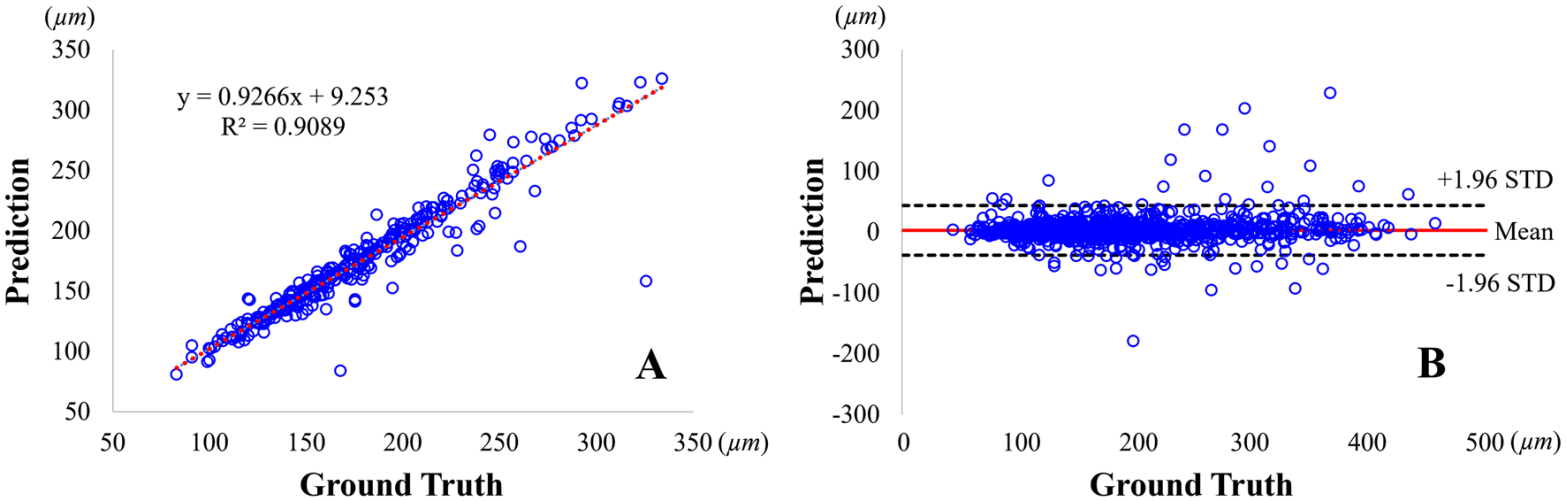
Comparison of mean FC thickness measurements between the ground truth and the proposed method, calculated on the held-out test set. The held-out test set comprised 1,362 FC images from 74 IVOCT pullbacks (UHCMC). In panel (**A**), the linear regression analysis demonstrates a remarkably high similarity (*R*^2^: 0.909) between the proposed method and the ground truth. Additionally, in the Bland–Altman analysis (panel **B**), the mean bias of FC thickness measurements was 2.95 ± 20.73 µm, with only a small number of cases (32 out of 1,362) exceeding the limits of agreement (indicated by black dotted lines). These findings indicate the absence of significant bias in the proposed method compared to the ground truth.

We found very good agreement of mean FC thickness measurements between our automated method and manual ground truth assessments (Fig. 7). The linear regression analysis showed an *R*^2^ of 0.909, indicating significant correlations between the ground truth and the proposed method (Fig. 7A). The mean bias of FC thickness measurements was only about 2.95 ± 20.73 µm in a Bland–Altman analysis, and most measurements (98%, 1330/1362) were included within the limits of agreement (Fig. 7B). These results support no significant bias of the proposed method as compared to the ground truth.

**Figure 8:**
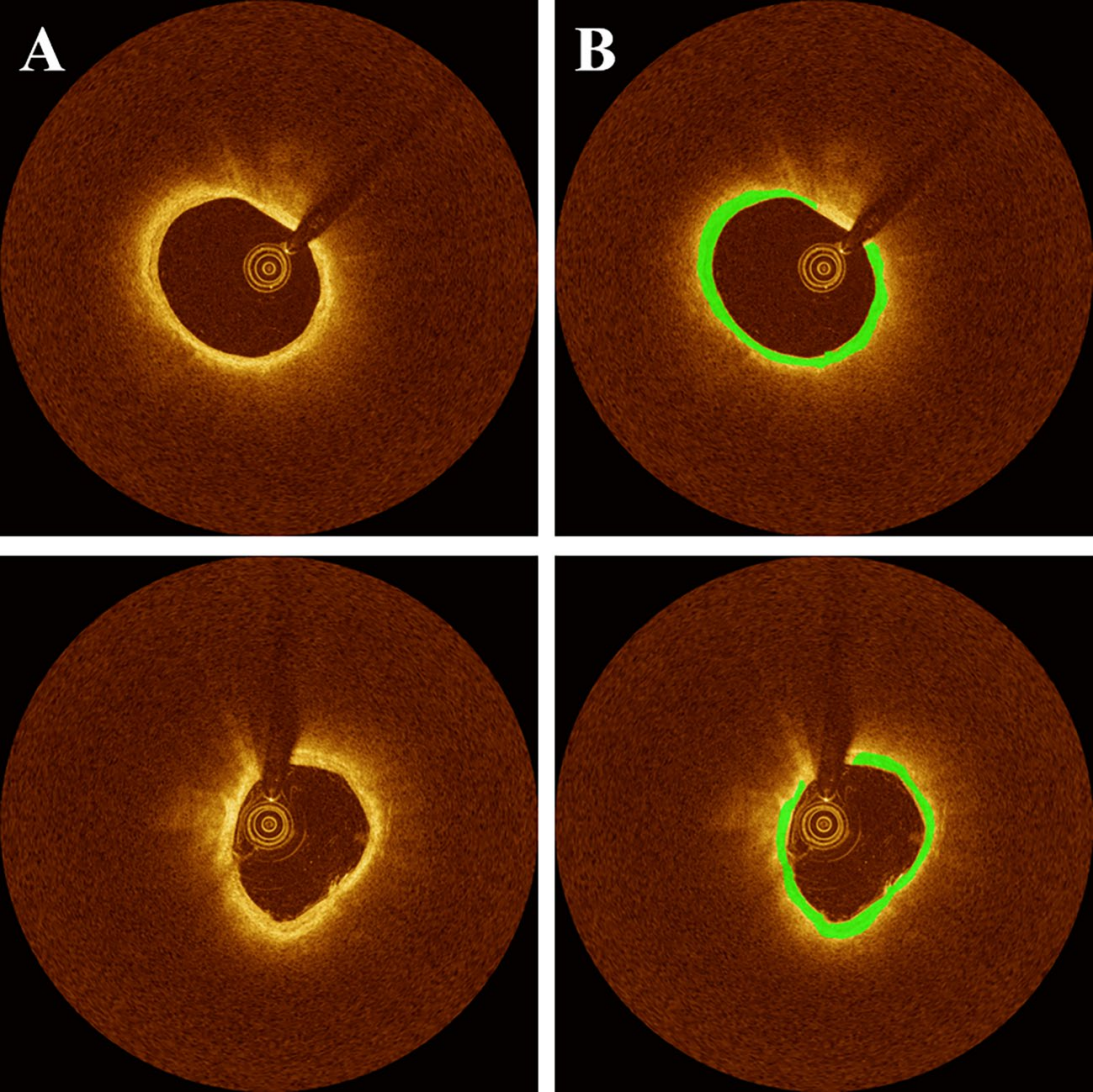
Reproducibility of FC assessment in scan-rescan IVOCT images as obtained from an untreated lesion in paired pre- and post-stenting IVOCT pullbacks. The panels include (**A**) Cartesian IVOCT image and (**B**) automated prediction. The top and bottom rows correspond to the first (pre-stenting) and second (post-stenting) scans, respectively. In this case, FC measurements between scans were as follows: FC thickness (114 µm/130 µm), FC arc angle (314°/321°), and FC area (1.76 mm^2^/1.49 mm^2^), indicating its excellent reproducibility. The color green represents FC plaque regions.

Our method demonstrated excellent reproducibility of automated FC segmentation in scan-rescan IVOCT images (Fig. 8). For this analysis, we utilized IVOCT images from paired pre- and post-stenting IVOCT pullbacks outside the stented region. We extracted 51 paired IVOCT images containing FCs, which were not included in the training or held-out test sets. Lesion attributes between pre- and post-stenting pullbacks were: average FC thickness (87.6 ± 38.6 µm/105.8 ± 33.9 µm), average FC arc angle (200.9 ± 128.0°/202.0 ± 121.1°), average FC area (1.04 ± 0.62 mm^2^/1.01 ± 0.56 mm^2^), and FC surface area (8.6 mm^2^/7.5 mm^2^). Additionally, the coefficients of variation between pre- and post-stenting pullbacks were very similar (Supplementary Table S4), indicating the strong reproducibility of our method.

**Figure 9:**
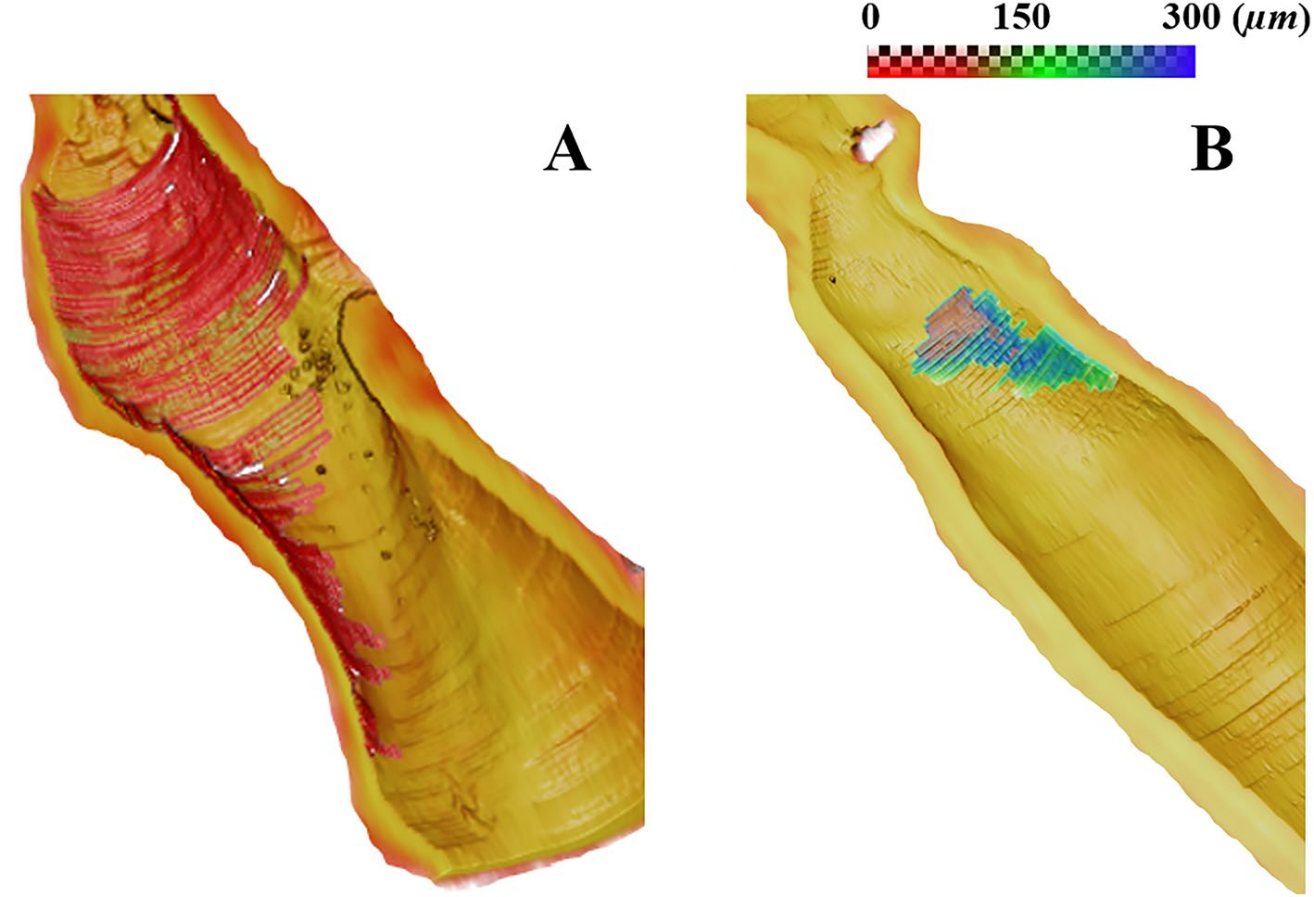
3D visualizations of FC thickness from representative IVOCT pullbacks with (**A**) large and (**B**) small lesions. Both lesions are by definition TCFAs because they have at least a point where the FC is under 65 µm. In the case of the large lesion, the FC had length = 28.2 mm, maximum angle = 271°, and surface area = 66.0 mm^2^. These values suggest vulnerability as compared to the small lesion with attributes of an FC length of 4.9 mm, a maximum angle of 109°, and a surface area of 4.2 mm^2^. For high-risk cases, clinicians may consider additional revascularization strategies such as a more aggressive statin treatment (see “Discussion”).

Figure 9 illustrates 3D visualizations of FC thickness in representative IVOCT pullbacks, featuring both large and small lesions (Fig. 9). For both type of lesions, the minimum thickness was below 65 µm, classifying them as TCFAs according to the standard definition. However, biomechainical considerations suggest that the larger lesion is likely to experience significantly greater strains compared to the smaller one, due to the additional support from surrounding normal tissues in the latter case. Consequently, the larger lesion may be more susceptible to rupture. The 3D visualizations were generated using Amira software, version 2023.2 (Thermo Fisher Scientific, Inc., Hillsboro, OR, USA).

## Discussion

In this study, we built on our previous studies of IVOCT image analysis^8,11–13,15,16,20,21,23,28,30–33^ and developed a fully-automated method for FC segmentation in IVOCT images using deep learning. The main findings are: (1) Transfer learning from a separate calcification segmentation task greatly reduced both the training time and the need for a large number of labeled training samples for deep learning. (2) The SegResNet network outperformed other deep learning networks, including U-Net, Attention U-Net, and nnU-Net. (3) Our method had a high generalizability evidenced by similar segmentation results on training (TRANSFORM-OCT) and held-out test (UHCMC) sets. (4).

Our method showed a good reproducibility for FC segmentation in scan-rescan IVOCT pullbacks. (5) The proposed method produced excellent results for FC segmentation while taking a reasonable amount of time (0.02 s per frame) to compute, implying that it could be a promising solution for both research and clinical applications. (6) Using our method, it is possible to create 3D FC thickness heatmaps and histograms of FC thickness of FC lesions, creating a broader characterization of lesion vulnerability.

Transfer learning enabled much faster training as compared to the traditional methods without transfer learning across all the networks used in this study. Transfer learning leverages prior knowledge gained while solving one task to solve a related new task^34^. In this study, prior knowledge was obtained from previous calcification segmentation^13,15,20,21^, and it was transferred to a related new task, which was FC segmentation. The whole training process can be made more efficient and generalizable by reusing elements of an algorithm and transferring the knowledge already held by a model. In addition, the sharing of knowledge between two different models can result in a more accurate and effective model overall. In our experiments, transfer learning achieved the desired performance in ~ 20% of the time. More importantly, with transfer learning, the network only required 60% of the training samples to reach the desired performance level. In our case, transfer learning was done from a similar task (calcification segmentation) to the target task (FC segmentation). We also tried pretraining using a conventional less related task (i.e., masked autoencoder). In our hands, using the more similar calcification task gave superior results.

Our method enabled excellent reproducibility of FC thickness measurement. From the experiments on the paired pre- and post-stenting IVOCT pullbacks, we found a very small bias and high similarity between those pullbacks (Fig. 8). IVOCT inevitably exhibits variations in plaque composition due to its reliance on the positioning of the catheter and guidewire, even when imaging the same lesion accurately. As a result, there were slight differences in the characteristics of FC plaque between the pre- and post-stenting pullbacks. Nonetheless, our automated analysis offered high repeatability, surpassing the potential variability introduced by different analysts, even with minimal user intervention. This suggests that our method is well-suited for large-scale research studies. Furthermore, with further improvements, it has the potential to be utilized clinically, assisting physicians in determining an appropriate stent landing zone.

We found an excellent generalizability of the proposed method for FC analysis using two large cohort data sets (i.e., TRANSFORM-OCT and UHCMC). Clinical research studies have mostly relied on single-center data, limiting their generalizability as they focus on local data despite the potential for larger datasets. Therefore, to generalize the clinical research methods (particularly deep learning applications), it is essential to use many data acquired from multiple sites. In our study, we created deep learning models using the TRANSFORM-OCT trial data, that mainly includes Italian patients, and validated its generalizability on the UHCMC data (Cleveland, OH, USA).

Automated segmentation of FC, particularly TCFA, in IVOCT holds the promise of advancing personalized medical treatments. The evolution of preventive and cardioprotective therapeutics over the past decade, such as P2Y12 antagonists, direct oral anticoagulants, proprotein convertase subtilisin kexin 9 (PCSK9) inhibitors, icosapent ethyl, and glucagon-like peptide 1 (GLP-1) agonists, underscores the necessity for personalized medicine approaches. These approaches aim to ensure that the most suitable treatment is administered to each patient in a cost-effective manner. The automated identification of FC tissue presents an opportunity to guide intensive therapies in clinical practice and enhance patient cohorts for evaluating the effectiveness of emerging novel therapeutics. Moreover, accurately determining TCFA could influence potential revascularization strategies. For instance, an additional stent might be considered to secure a high-risk lesion, and a more aggressive statin treatment could also be prescribed alongside stenosis. Assessing plaque changes between baseline and follow-up pullbacks has the potential to facilitate mechanistic studies in drug development. Furthermore, identifying high-risk IVOCT characteristics, including TCFA, has the potential to contribute valuable insights to other imaging modalities.

This study has identified some limitations. First, our method occasionally generated inaccurate results, particularly in the presence of side branches or mixed plaque (refer to Supplementary Fig. S2). However, it is important to note that such instances were infrequent and easily correctable. Second, despite utilizing a large dataset for this study, there is room for further improvement in future investigations by employing even larger datasets. Some scenarios, such as cases involving significant side branches and thrombus, were not fully represented in the training data. The implementation of active learning or human-in-the-loop learning, where cases are segmented, corrected, and reintegrated into the training dataset, holds the potential to enhance performance. Third, the observed performance differences among networks suggest that more advanced deep learning approaches could lead to further improvements. Fourth, the proposed method has not been validated using an external validation dataset. As a result, there may be discrepancies in performance when applied to other datasets. In subsequent studies, we intend to enhance segmentation performance by incorporating larger datasets and validate the robustness and reproducibility across multiple institutions worldwide.

In conclusion, we developed a fully automated, deep learning FC segmentation and measurement methods for IVOCT images. Using multi-center datasets, we performed rigorous evaluations and demonstrated excellent performance, generalizability, and reproducibility. We believe that this method will prove useful for various research applications and may even play a role in future treatment planning, especially when one wants to avoid placing a stent edge over a lipidic lesions with a vulnerable FC.

### Supplementary Information

Supplementary Information.

## Data Availability

The datasets generated and/or analyzed during the current study are available from the corresponding author upon reasonable request.
