# Supplementary material for: Deep learning segmentation of fibrous cap in intravascular optical coherence tomography images
Source: Sci Rep. 2024 Feb 22;14:4393. doi: 10.1038/s41598-024-55120-7 (PMC10884035; doi:10.1038/s41598-024-55120-7)
Supplement: Supplementary file 1 — Supplementary Information. [file 41598_2024_55120_MOESM1_ESM.docx]

**Supplementary Materials**

Table S1 Training details of deep learning networks, including U-Net, Attention U-Net, nnU-Net, and SegResNet.

|  | U-Net | Attention U-Net | nnU-Net | SegResNet |
| --- | --- | --- | --- | --- |
| Input size | 256x448 | 256x448 | 256x448 | 256x448 |
| Kernel size | 3x3 | 3x3 | 3x3 | 3x3 |
| Normalization | Instance Norm | Instance Norm | Instance Norm | Group Norm |
| Dropout | 0.2 | 0.2 | 0.2 | 0.2 |
| Deep supervision | - | - | Off | - |
| Initial filters | - | - | - | 16 |
| Activation function | ReLU | ReLU | leakyReLU | ReLU |
| Optimization | AdamW | AdamW | AdamW | AdamW |
| Loss function | Dice | Dice | Dice | Dice |


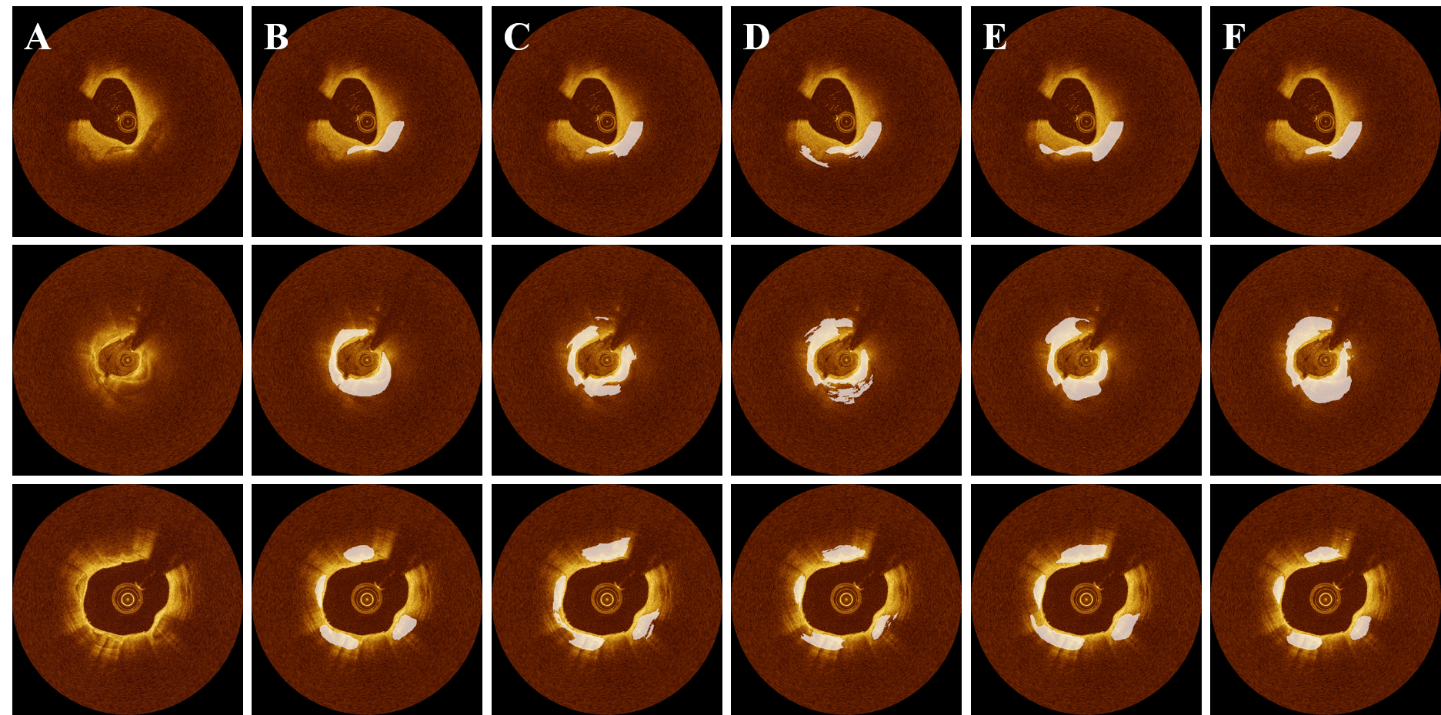


Fig. S1. Automated calcium segmentation results obtained using four different deep learning models. Panels include (A) original image, (B) ground truth, (C) U-Net, (D) Attention U-Net, (E) nnU-Net, and (F) SegResNet. Each row represents different instances of IVOCT images. Overall, SegResNet exhibited the most favorable segmentation results among all the networks employed. The color white indicates areas of calcification.

Table S2 Quantitative performance metrics of calcification segmentation, including PPV, NPV, sensitivity, specificity, accuracy, and Dice coefficient. The networks were trained using existing labeled calcium IVOCT dataset. Among all the networks, SegResNet demonstrated the highest values for PPV, sensitivity, specificity, accuracy, and Dice coefficient, indicating superior performance in calcium segmentation.

|  | PPV (%) | NPV (%) | Sensitivity (%) | Specificity (%) | Accuracy (%) | Dice |
| --- | --- | --- | --- | --- | --- | --- |
| U-Net | 77.2 | 97.4 | 76.5 | 97.5 | 95.4 | 0.768 |
| Attention U-Net | 76.3 | 97.2 | 74.6 | 97.4 | 95.2 | 0.755 |
| nnU-Net | 67.4 | 98.6 | 87.4 | 95.3 | 94.5 | 0.761 |
| SegResNet | 79.2 | 97.8 | 80.6 | 97.7 | 96.0 | 0.799 |

The pretrained models for transfer learning yielded reasonable segmentation results for calcification. In the Supplementary Figure 1, the automated calcification predictions from the four different networks on the validation set are depicted. Among them, the Attention U-Net exhibited the largest segmentation errors across most instances, irrespective of the calcification phenotype, and achieved the lowest sensitivity (74.6%) and Dice coefficient (0.755). The U-Net produced similar results. The nnU-Net tended to slightly overestimate coronary calcification, resulting in the lowest PPV (67.4%) despite its high sensitivity (87.4%). On the other hand, the SegResNet demonstrated the most reliable segmentation outcomes, with the highest PPV and Dice coefficient. The Supplementary Table 1 presents the quantitative metrics for calcification segmentation across the four different deep learning networks.

Table S3 Mean quantitative performance metrics of FC segmentation over five folds, comparing results obtained with and without transfer learning (TL). As described in the Results, the application of transfer learning did not yield a significant improvement in segmentation performance. However, it notably reduced the training time for all the networks employed.

|  | PPV (%) | NPV (%) | Sensitivity  (%) | Specificity (%) | Accuracy  (%) | Dice |
| --- | --- | --- | --- | --- | --- | --- |
| U-Net wo TL | 84.3±1.7 | 98.8±0.1 | 79.6±0.3 | 99.1±0.1 | 98.1±0.0 | 0.819±0.006 |
| U-Net w TL | 84.0±1.8 | 98.9±0.1 | 80.2±0.1 | 99.1±0.1 | 98.1±0.0 | 0.820±0.009 |
| Attention U-Net wo TL | 79.2±1.0 | 98.7±0.2 | 77.3±1.5 | 98.8±0.0 | 97.7±0.1 | 0.782±0.003 |
| Attention U-Net w TL | 81.2±0.6 | 98.9±0.2 | 80.1±4.9 | 98.9±0.2 | 97.9±0.1 | 0.806±0.022 |
| nnU-Net wo TL | 80.7±1.5 | 99.3±0.0 | 87.2±0.3 | 98.8±0.1 | 98.2±0.0 | 0.838±0.009 |
| nnU-Net w TL | 77.2±0.3 | 99.5±0.1 | 91.4±1.5 | 98.5±0.1 | 98.1±0.0 | 0.837±0.008 |
| SegResNet wo TL | 84.9±1.8 | 99.0±0.1 | 82.8±0.3 | 99.2±0.1 | 98.3±0.0 | 0.838±0.008 |
| SegResNet w TL | 84.2±1.8 | 99.1±0.0 | 85.0±0.3 | 99.1±0.1 | 98.3±0.1 | 0.846±0.011 |

Table S4 Lesion attributes between pre- and post-stenting pullbacks, including average FC thickness, average FC arc angle, average FC area, and FC surface area. As described in the Results, our method demonstrated excellent reproducibility in automated FC segmentation. STDV represents the standard deviation, and COV represents the coefficient of variance.

|  | Average  FC thickness (*µm*) | | Average  FC arc angle (°) | | Average  FC area (*mm^2^*) | | FC surface area (*mm^2^*) | |
| --- | --- | --- | --- | --- | --- | --- | --- | --- |
|  | Pre | Post | Pre | Post | Pre | Post | Pre | Post |
| Mean | 87.6 | 105.8 | 200.9 | 202.0 | 1.04 | 1.01 | 8.6 | 7.5 |
| STDV | 38.6 | 33.9 | 128.0 | 121.1 | 0.62 | 0.56 | - | - |
| COV | 0.44 | 0.32 | 0.64 | 0.60 | 0.59 | 0.56 | - | - |


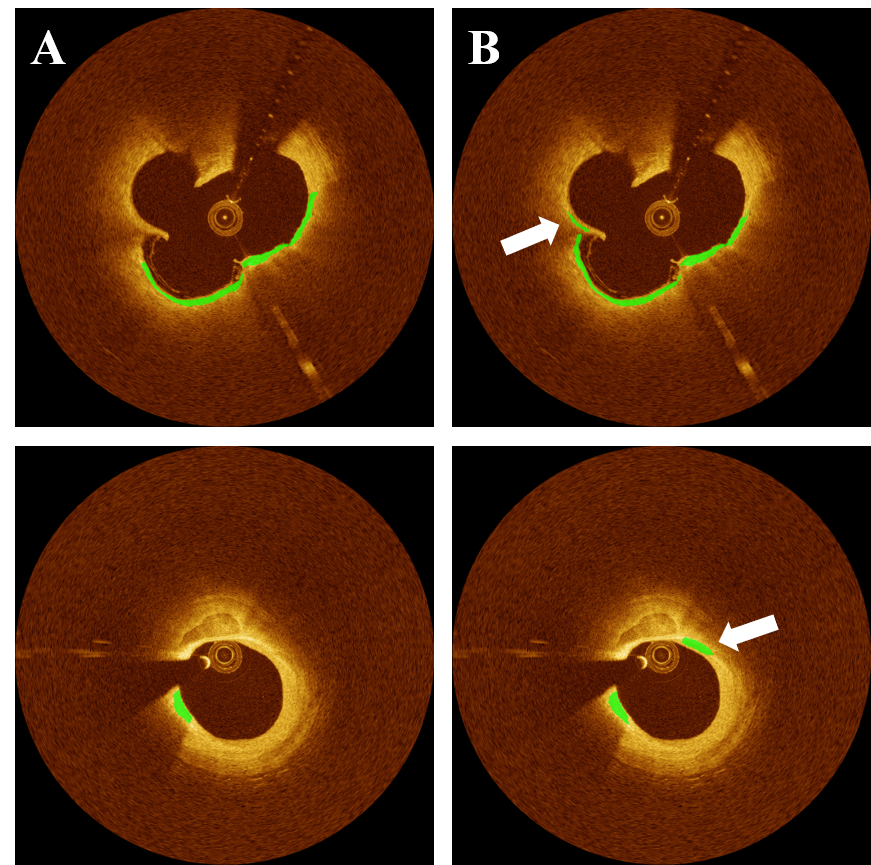


Fig. S2. Illustration of false prediction instances. The panels are (A) manual annotation and (B) automated prediction. In the top example, the proposed method exhibited a false prediction, possibly attributed to the presence of a significant side branch. In the bottom example, the proposed method demonstrated a false prediction concerning a mixed plaque. The false predictions are highlighted with white arrows.
